# A flexible transparent gas barrier film employing the method of mixing ALD/MLD-grown Al_2_O_3_ and alucone layers

**DOI:** 10.1186/s11671-015-0838-y

**Published:** 2015-03-14

**Authors:** Wang Xiao, Duan Ya Hui, Chen Zheng, Duan Yu, Yang Yong Qiang, Chen Ping, Chen Li Xiang, Zhao Yi

**Affiliations:** State Key Laboratory on Integrated Optoelectronics, College of Electronic Science and Engineering, Jilin University, Jilin, 130012 China

**Keywords:** Thin film encapsulation, Water vapor transmission rate, Molecular layer deposition, Low-temperature atomic layer deposition

## Abstract

Atomic layer deposition (ALD) has been widely reported as a novel method for thin film encapsulation (TFE) of organic light-emitting diodes and organic photovoltaic cells. Both organic and inorganic thin films can be deposited by ALD with a variety of precursors. In this work, the performances of Al_2_O_3_ thin films and Al_2_O_3_/alucone hybrid films have been investigated. The samples with a 50 nm Al_2_O_3_ inorganic layer deposited by ALD at a low temperature of 80°C showed higher surface roughness (0.503 ± 0.011 nm), higher water vapor transmission rate (WVTR) values (3.77 × 10^−4^ g/m^2^/day), and lower transmittance values (61%) when compared with the Al_2_O_3_ (inorganic)/alucone (organic) hybrid structure under same conditions. Furthermore, a bending test upon single Al_2_O_3_ layers showed an increased WVTR of 1.59 × 10^−3^ g/m^2^/day. However, the film with a 4 nm alucone organic layer inserted into the center displayed improved surface roughness, barrier performance, and transmittance. After the bending test, the hybrid film with 4 nm equally distributed alucone maintained better surface roughness (0.339 ± 0.014 nm) and barrier properties (9.94 × 10^−5^ g/m^2^/day). This interesting phenomenon reveals that multilayer thin films consisting of inorganic layers and decentralized alucone organic components have the potential to be useful in TFE applications on flexible optical electronics.

## Background

Organic electronics is an emerging technology that has potential uses in highly efficient lighting, super-bright displays, novel photovoltaic devices, and integrated smart systems [1-3]. Furthermore, it offers promising opportunities for the development of new products that utilize the special features of organic electronics such as flexibility, bendability, and transparency [4-6]. However, one major impediment to the mass production of organic devices is insufficient product lifetimes caused by their inclination to stop functioning when exposed to water vapor, oxygen, and other detrimental compounds present in air. Encapsulation layer, also known as barrier film, is a necessary and often overlooked part of the organic device architecture. Furthermore, polymer substrates, often used in flexible devices, provide better flexibility and toughness properties, but possess insufficient barrier properties against water vapor and oxygen permeation [7]. Since oxide films have to be of high quality to provide superior barrier performance, atomic layer deposition (ALD) is being pursued as an alternative to traditional chemical and physical vapor deposition methods. Reducing the number of defects (pinholes, grain boundaries, etc.) can reduce the layer thickness and/or number of layers required to achieve the required water vapor transmission rates (WVTR, g/m^2^/day). Recently, this type of thin film encapsulation (TFE) has attracted great attention in order to overcome the air-sensitive issue [8-10]. The inorganic/organic encapsulation method based on ALD and molecular layer deposition (MLD), respectively, has demonstrated better barrier performance and mechanical properties than single inorganic layers [11-13]. On the one hand, the organic layer could potentially decouple any defects and prolong the permeation path, leading to lower WVTR values [14,15]. On the other hand, single inorganic encapsulation films are brittle in general, but the hybrid inorganic/organic structure reduces the internal stress of inorganic films generally improving flexibility [16,17].

It is therefore important to consider the development of high-barrier functionalities as well as the mechanical properties of TFE samples. In this study, samples with Al_2_O_3_ (ALD) or alucone (MLD) layers were grown and characterized. All encapsulation films were deposited at a low temperature of 80°C [18,19]. We investigated single Al_2_O_3_ films with Al_2_O_3_/alucone hybrid laminate before and after a bending test. The gas barrier and mechanism performances were both optimized [20] upon Al_2_O_3_ samples incorporating a 4-nm transparent organic component of the same nominal thickness. From this analysis, some important insights were determined, demonstrating that the performance of TFE with hybrid inorganic-organic structure could be optimized by prudent selection of certain design parameters.

## Methods

**Figure 1:**
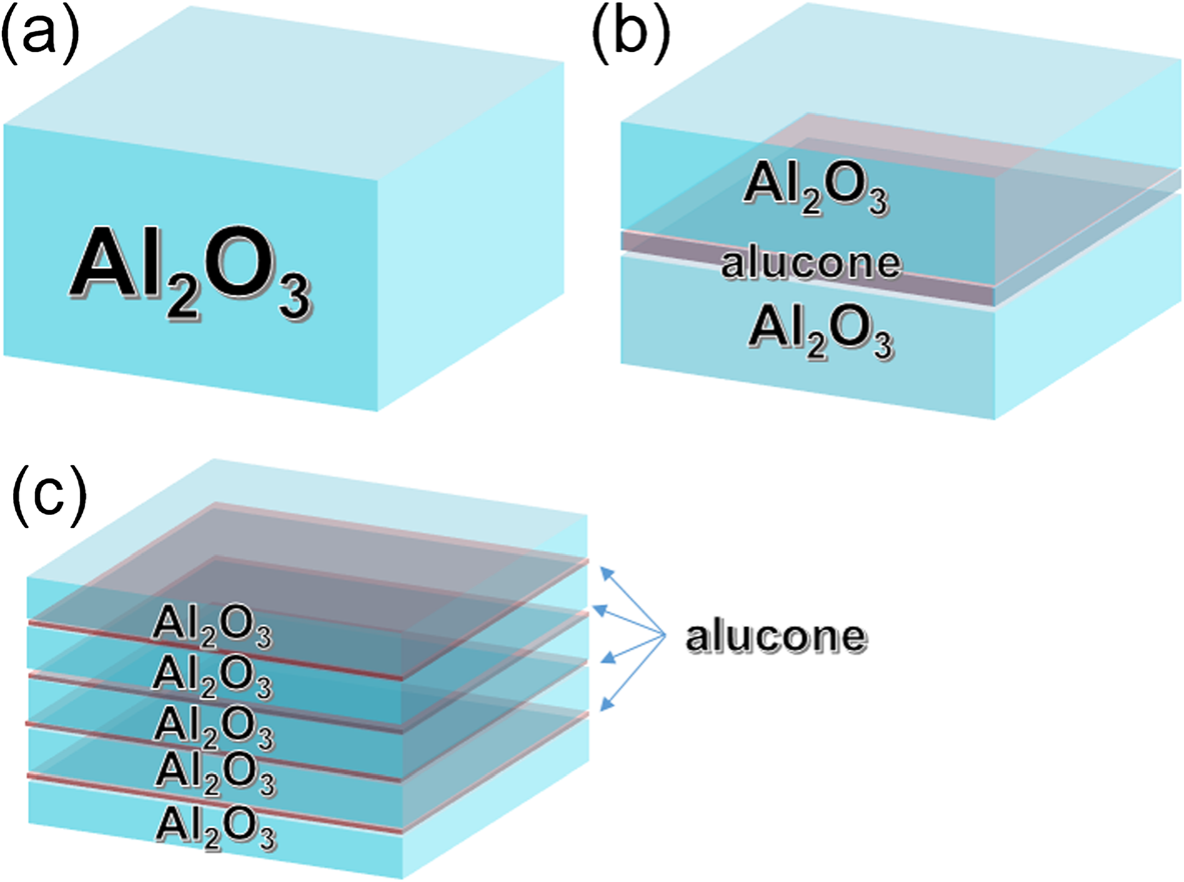
**A schematic diagram of prepared TFE structures. (a)** Film A: Al_2_O_3_ 50 nm. **(b)** Film B: Al_2_O_3_/alucone/Al_2_O_3_: 23/4/23 nm. **(c)** Film C: Al_2_O_3_/alucone/Al_2_O_3_/alucone/Al_2_O_3_/alucone/Al_2_O_3_/alucone/Al_2_O_3_ 9/1/9/1/9/1/9/1/9 nm.

In the experiments, we fabricated a group TFE consisting of three different thin films. All films have nominal thicknesses of approximately 50 nm. As shown in Figure 1, film A was a 50 nm Al_2_O_3_ inorganic film. Films B and C consisted of approximately 46 nm Al_2_O_3_ and 4 nm alucone. For film B, 4 nm alucone was in the center of the hybrid film (23/4/23 nm). However, the alucone layer was divided into four equal parts in film C (9/1/9/1/9/1/9/1/9 nm). Both Al_2_O_3_ and alucone thin films were deposited by a LabNano 9100 ALD system (Ensure Nanotech Inc., Beijing, China) at 80°C, and all pipes were heated to 120°C, while the pressure in the reaction chamber was 1.5 × 10^0^ Pa.

Table 1 summarizes the film deposition parameters during the ALD process. Al(CH_3_)_3_ (trimethylaluminum or TMA, Sigma Aldrich, St. Louis, MO, USA) and deionized water were prepared as precursors for Al_2_O_3_ inorganic layer. During the growth process, high-purity N_2_ (flow rate = 20 sccm) was used as carrier gas for these precursors. One reaction cycle included the following: 0.02 s TMA dose, 30 s nitrogen purge, 0.02 s H_2_O dose, and 30 s nitrogen purge. This sequence was repeated to obtain the desired thicknesses. For alucone organic layer, TMA and HO-(CH_2_)_2_-OH (ethylene glycol or EG, Sigma Aldrich) were reactants grown under identical conditions. Before the deposition process, EG was preheated to 95°C to increase its vapor pressure [21]. The timing sequence was as follows: 0.02 s TMA dose, 30 s nitrogen purge, 0.07 s EG dose, and 120 s nitrogen purge. The growth mechanism for each type of film has been described previously [22]. WVTR measurements were carried out to test the barrier performance of the films through the calcium (Ca) corrosion method. The amount of water vapor permeating through the film was estimated with the following formula [11]:

$$ \mathrm{WVTR}\left[\mathrm{g}/\mathrm{m}2/\mathrm{day}\right]=\hbox{-} n\times \delta \mathrm{C}\mathrm{a}\times \rho \mathrm{C}\mathrm{a}\times \frac{d}{\mathrm{dt}}\left(\frac{1}{R}\right)\times \frac{\mathrm{M}\left(\mathrm{H}2\mathrm{O}\right)}{\mathrm{M}\left(\mathrm{C}\mathrm{a}\right)}\times \frac{\mathrm{Ca}\_\mathrm{Area}}{\mathrm{Window}\_\mathrm{Area}} $$

**Table 1:** **The thin film deposition parameters for the ALD process**

**Film**	**TMA pulse time (s)**	**N**_**2**_ **purge time (s)**	**H**_**2**_ **O/EG pulse time (s)**	**N**_**2**_ **purge time (s)**	**Temperature (°C)**	**Pressure (Pa)**	**Carrier gas**
Al_2_O_3_	0.02	30	0.02	30	80	1.5 × 10^0^	N_2_
Alucone	0.02	30	0.07 (preheated to 95°C)	120	80	1.5 × 10^0^	N_2_

Ca_Area/Window_Area represents the effective testing area to mask window area ratio. In this experiment, Ca_Area/Window_Area is equal to 1. The root-mean-square roughness (RMS) and other surface features of the films were measured with a Veeco AFM. The thickness and refractive index of the thin films deposited on clean Si substrate were measured using a J.A. Woolam variable-angle spectroscopic ellipsometer (J.A. Woollam Co. Inc., Lincoln, NE, USA). The electrical characteristics of the devices were measured with an Agilent 2920 source meter (Agilent Technologies, Inc., Santa Clara, CA, USA) at room temperature.

## Results and discussion

**Figure 2:**
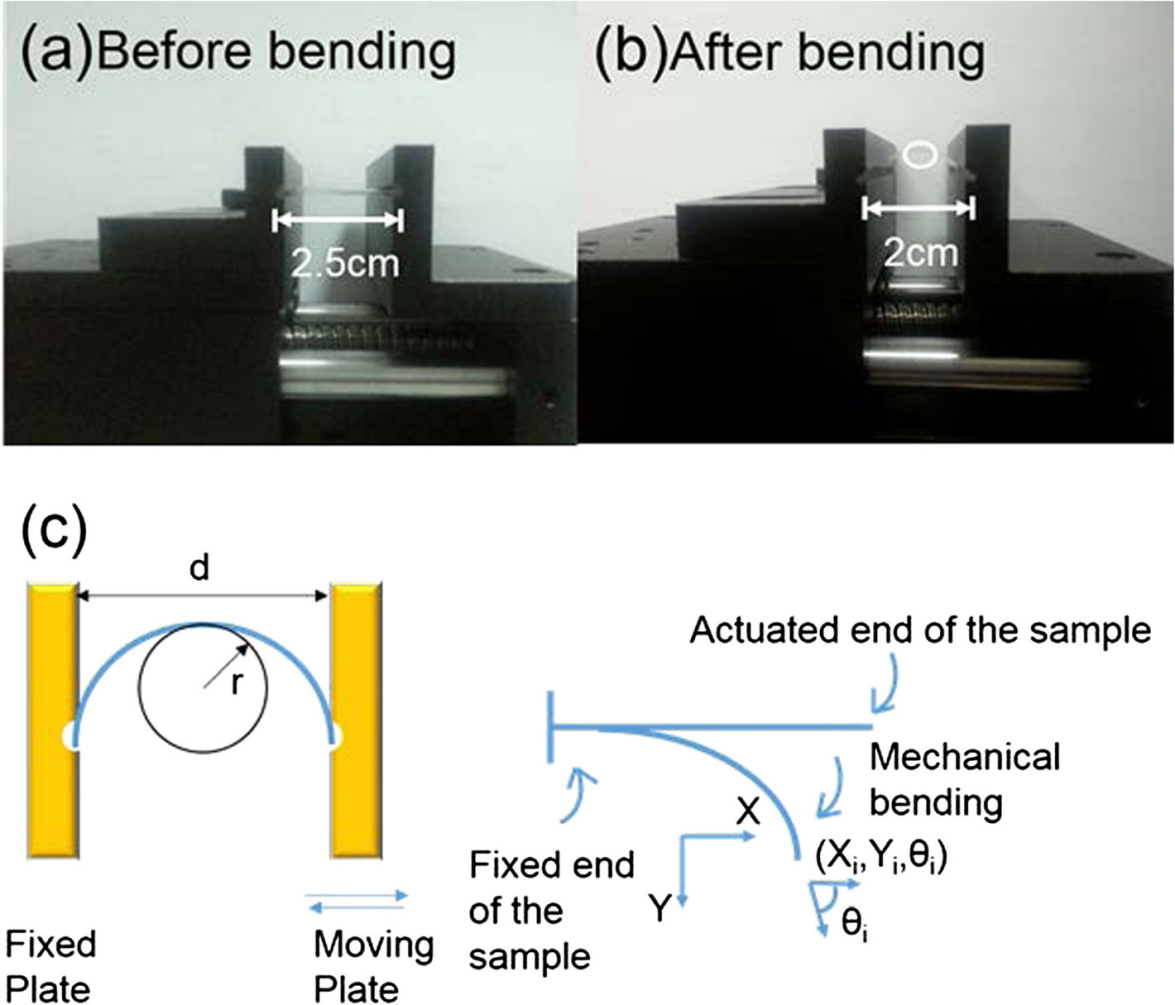
**The photo and schematic diagram of the bending device. (a)** The initial state. **(b)** The final state. **(c)** The schematic diagram of the bending test.

Table 2 summarizes some surface characteristics after thin film deposition by ALD at 80°C, including film thickness, normalized growth rate, RMS, and water contact angle. Previous research by Dameron et al. reported that the alucone organic films showed a growth rate of 4 Å/cycle at 85°C, much faster than approximately 1 Å/cycle for Al_2_O_3_ at 80°C [23]. Here, a similar MLD deposition rate was achieved at 3.8 Å/cycle at 80°C, which further indicates that MLD alucone is typically a bifunctional monomer for fast stepwise condensation polymerization and yield completely organic films. Figure 2a,b shows the setup of the device for the investigation of the mechanism behavior of TFE under oscillatory bending. A square film sample was loaded between parallel plates. One of the plates was mounted on an oscillatory driven stepper motor. The number of revolutions performed by the motor controls the frequency. In the middle of the bend, the lowest radius of curvature *r* and the largest tensile strain at the *y*-axis (Figure 2c) were determined by the distance between parallel plates. In this study, the distance was fixed at approximately 2 mm and the radius of curvature *r* was 1.05 mm. The white circle in Figure 2b marks the maximum curved position where the AFM images were taken.

**Table 2:** **A summary of the surface film characteristics after deposition by ALD/MLD**

**Film code**	**Thickness (nm)**	**Normalized growth rate (Å/per cycle)**	**RMS roughness (nm)**	**Contact angle (°)**
A	52.137 ± 0.034	0.947 ± 0.001	0.503 ± 0.011	65.3 ± 3.7
B	53.693 ± 0.156	1.161 ± 0.003	0.492 ± 0.002	95.1 ± 3.3
C	54.956 ± 0.067	1.153 ± 0.001	0.465 ± 0.012	86.5 ± 1.4

**Figure 3:**
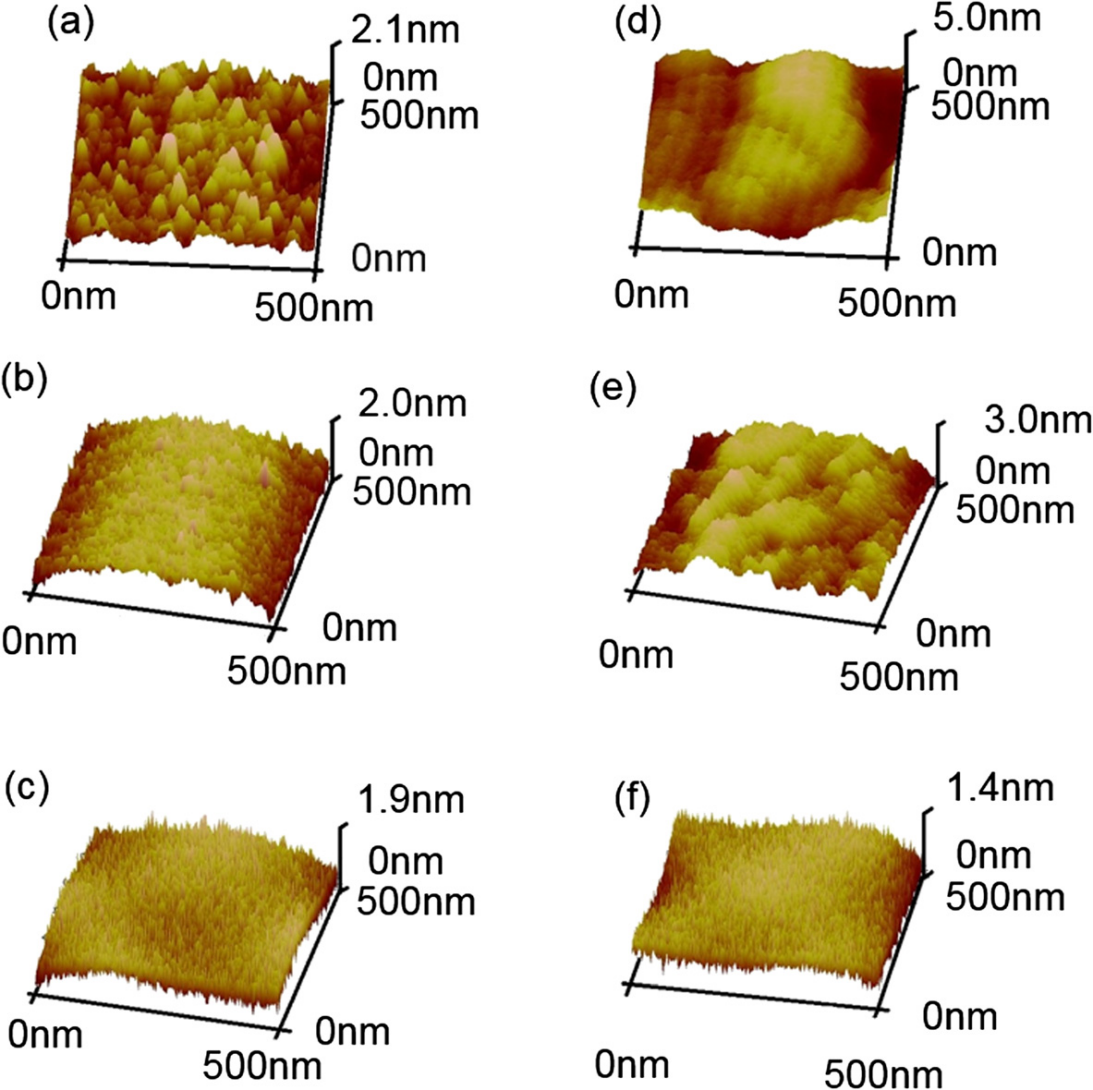
**Atomic force microscope (AFM) images on clean PET substrate. (a-c)** Film A, B, C before the bending test. **(d-f)** Film A, B, C after the bending test.

**Figure 4:**
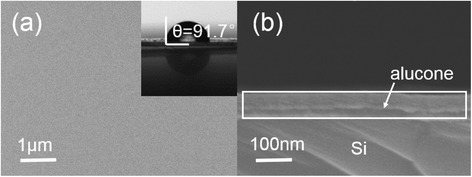
**Scanning electron microscope (SEM) images of film B on clean Si substrate. (a)** The surface image of film B; the insert is water contact angles. **(b)** The cross-sectional image of film B.

Figure 3 shows the surface topography and roughness of all thin films measured with AFM before and after the bending test. Before the bending test, a RMS of 0.503 ± 0.011 nm, 0.492 ± 0.002 nm, and 0.465 ± 0.012 nm was obtained for films A, B, and C, respectively. These values were almost equal to the bare PET substrate (RMS = 0.522 ± 0.007 nm). The highly conformal thin films were attributed to the use of ALD and MLD techniques. The slight negative trend from film A to film C might be due to the organic layer smoothing the surface [24]. However, film A (the single Al_2_O_3_ inorganic layer) exhibited an increase in RMS of 1.210 ± 0.034 nm after the bending test, while film B-C presented still lower values of 0.761 ± 0.021 and 0.339 ± 0.032 nm, respectively. We deduce that during the bending process, the alucone organic layer served as a stress buffer layer and might account for the lower RMS values. In the case of film C, the internal stress of Al_2_O_3_ layer was alleviated the most from separated organic layers [25]. Figure 4 shows the SEM image of film B deposited by ALD/MLD at 80°C. The Al_2_O_3_/alucone hybrid film appeared to be homogeneous with a smooth surface. The contact angle was found to be 95.1 ± 3.3° and 86.5 ± 1.4° for film B and C, respectively. This is higher than the value of single Al_2_O_3_ films (65.3 ± 3.7°). This phenomenon was attributed to the surface of the hybrid film being smoother than Al_2_O_3_, and this could be evidence for the possible dependence of the contact angle on the surface morphology.

**Figure 5:**
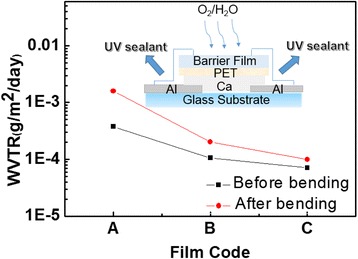
**The calculated WVTR changes for different film codes before and after the bending test.** The insert is the schematic diagram of the Ca test.

**Figure 6:**
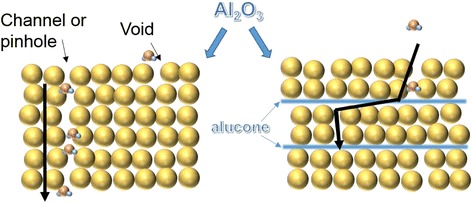
**The schematic diagram of water vapor permeation for Al**_**2**_ **O**_**3**_ **and Al**_**2**_ **O**_**3**_ **/alucone hybrid film in air.**

To evaluate the permeability of Al_2_O_3_/alucone films as a water diffusion barrier, we studied the films before and after the bending test. The Ca sample wafers (Glass/Ca (200 nm)/Al (100 nm)) were deposited by thermal evaporation equipment at 5 × 10^−4^ Pa without breaking the vacuum and were then transferred to a glove box. The area of Ca thin films was 1 × 1 cm^2^. The barrier films deposited by ALD/MLD on clean PET substrates were adhered to the Ca samples by UV sealant as shown in the inset of Figure 4 [26]. The calculated WVTR changes for different films before and after the bending test were shown in Figure 5. Before the bending test, the WVTR was found to be 3.77 × 10^−4^ g/m^2^/day (film A), 1.06 × 10^−4^ g/m^2^/day (film B), and 7.1 × 10^−5^ g/m^2^/day (film C). This was attributed to the fact that the alucone organic layer increases the permeation path for water vapor in the hybrid structure. It also reacts with the water vapor, decreasing the diffusion speed [11,27]. Figure 6 illustrates the water vapor permeation process for different thin film structures. With a 4-nm-thick alucone organic component divided into four equal layers, a 40% decrease in WVTR was obtained when comparing films B and C. As confirmed by our previous research [20], the increased proportion of Al_2_O_3_ in the hybrid structure leads to an improved barrier performance. However, after the bending test, the barrier performances demonstrated evidence of different degrees of damage. A notable increase from 3.77 × 10^−4^ to 1.59 × 10^−3^ g/m^2^/day in WVTR was obtained for film A, while a more subtle increase from 9.94 × 10^−5^ to 7.1 × 10^−5^ g/m^2^/day was achieved for film C. The results indicate that internal alucone organic layers improve flexibility under the same thicknesses. When the alucone organic layer was separated into separate layers, it leads to a more even distribution of stress in the laminates and reduced destruction [25].

**Figure 7:**
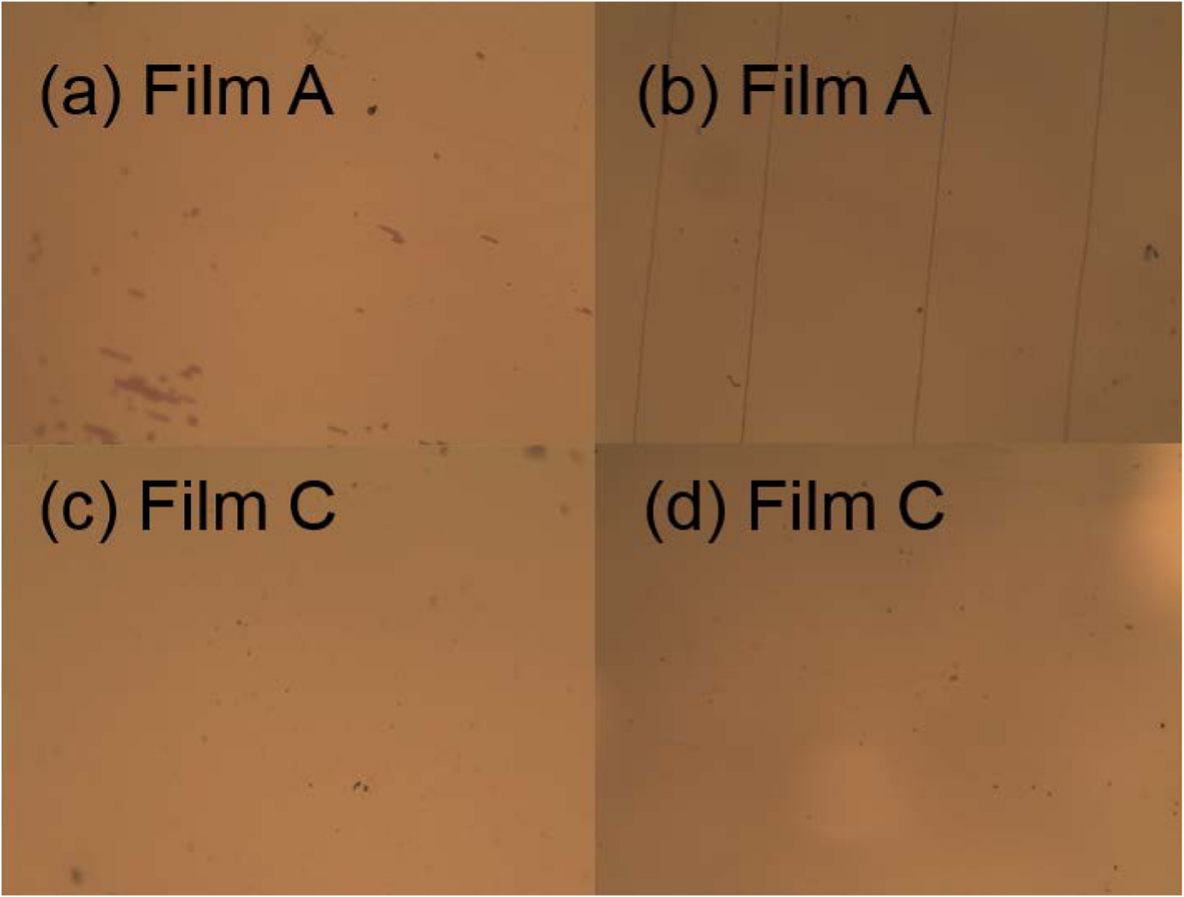
**The microscopic pictures for film A and film C before and after the bending test. (a,b)** were taken from the surface of film A; **(c,d)** were from film C.

In order to demonstrate the effect of the bending test on different films, we took some microscopic pictures for film A and film C in contrast to see if there were some damage after the bending test. As can be clearly seen from Figure 7(a),(c) were taken from the surface of films A and C, respectively, before the bending test. There was no obvious difference between the two films. Figure 7b was film A after the bending test; we could clearly see some parallel stripes. On the contrary, there was no such phenomenon in (d) taken from film C after the bending test. We believe it was the alucone organic layer that served as buffer layer easing the stress under bending test. The cracks from the surface of film A were an evidence for the raising RMS and WVTR values.

**Figure 8:**
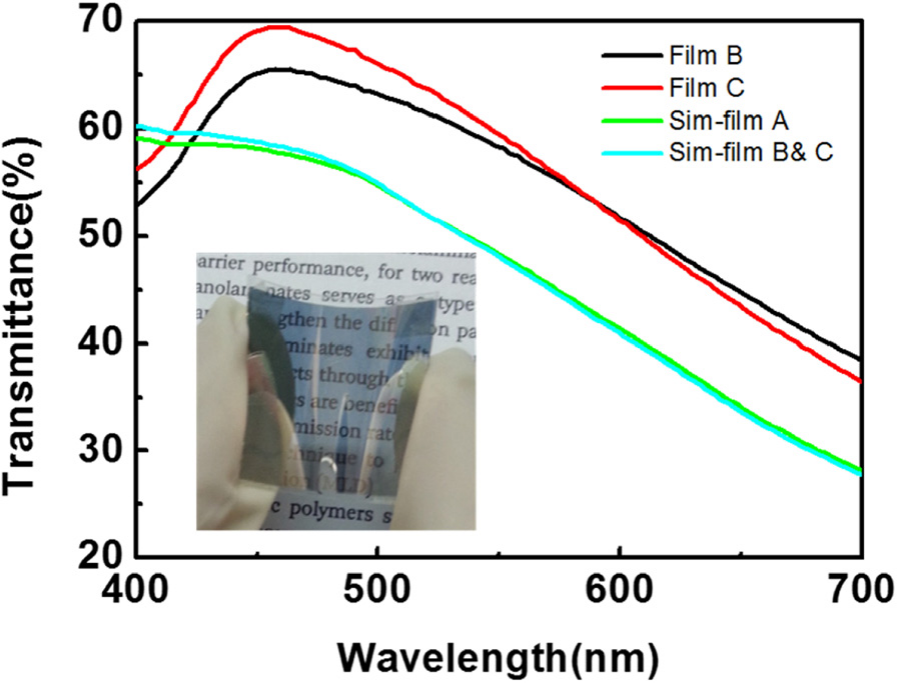
**Experimental and simulated results of transmittance of the films based on Alq**_**3**_ **(50 nm)/Ag (20 nm)/TFE/Air structure.** The insert is a photo of Alq_3_ (50 nm)/Ag (20 nm)/film C structure on PET substrate.

Finally, the optical properties of TFE samples were measured as well as simulated. Figure 8 shows the simulated and experimental transmittance of Alq_3_ (50 nm)/Ag (20 nm)/TFE/air structures on a PET substrate before carrying out the bend test. For all tests, no obvious change in transmittance was observed, even after 600 iterations of the bending test. Film C (maximum transmittance of 69%) showed a slightly higher transmittance at the region of 400-580 nm compared with film B (maximum transmittance of 65%) (Figure 8). In addition, simulated results predicated that the hybrid film would have similar transmittance values with the single Al_2_O_3_ film over the whole visible region. This optical characteristic is beneficial due to the fact that alucone has superior photo permeability [25] and this may potentially be useful for the TFE design in top emitting organic light devices or organic photovoltaics.

## Conclusions

In summary, a hybrid ALD/MLD deposition technique has been used at a low temperature of 80°C in order to fabricate multiple stacked layers of Al_2_O_3_/alucone thin film encapsulations. Single Al_2_O_3_ film and Al_2_O_3_/alucone hybrid films have been investigated for the potential usage on flexible PET substrates. By introducing a 4 nm alucone organic layer inside and separating them into four equal layers inside the TFE structure, the hybrid structure delivers a considerably lower gas permeation (WVTR = 9.94 × 10^−5^ g/m^2^/day), higher flexibility, and transparency performance. This information will be useful for encapsulation structure engineering, to eventually enable optimal design of organic electronics.

## Abbreviations

ALD: atomic layer deposition
TFE: thin film encapsulation
WVTR: water vapor transmission rate
MLD: molecular layer deposition
EG: ethylene glycol
AFM: atomic force microscopy
SEM: scanning electron microscopy
